# Parasitic omental ovarian dermoid tumour mimicking an adnexal mass: A report of two very unusual cases

**DOI:** 10.4274/tjod.87259

**Published:** 2015-12-15

**Authors:** Hüseyin Çağlayan Özcan, Mete Gürol Uğur, Reyhan Gündüz, Zehra Bozdağ, İrfan Kutlar

**Affiliations:** 1 Gaziantep University Faculty of Medicine, Department of Obstetrics and Gynecology, Gaziantep, Turkey; 2 Gaziantep University Faculty of Medicine, Department of Pathology, Gaziantep, Turkey

**Keywords:** Parasitic dermoid cyst, omentum, pelvic mass

## Abstract

The differential diagnosis of cystic adnexal masses includes various pathologies, some placed extragonadally. Herein, we present two different cases of omental ovarian dermoid tumours that were diagnosed using ultrasonography and removed with surgery. The greater part of the omental teratomas appear to have developed from self-amputation of cysts in the ovary, followed by their re-implantation into the omentum. Omental teratomas can be located in the pelvis, where they might be mistaken for an adnexal mass, an upper abdominal mass, or a periumbilical mass. The location of omental teratomas might slightly change from one examination to another. In such cases, preoperative diagnostic imaging methods may not provide adequate information to physicians. Gynecologists should always keep in mind the possibility of intraabdominal ovarian parasitic cystic teratomas in the differential diagnosis of suspicious adnexal masses during surgery. Awareness among gynecologic surgeons of such masses may help prevent misdiagnosis, delayed surgery, or the use of wrong surgical approaches.

## INTRODUCTION

In contrast to dermoid cysts, which are also known as mature cystic teratomas and represent a frequently observed type of ovarian tumour, parasitic ovarian dermoid tumours tend to be highly uncommon, accounting for only 0.4% of teratomas in the ovaries^(1)^. Although teratomas are rarely encountered in the omentum, parasitic dermoid cysts are often localized in the omentum^(1)^. Lebert first described omental dermoid cysts in 1734^(2)^. These cysts are usually observed among reproductive-age women, although they can also be found in young girls and elderly women in certain cases^(1)^. An examination of the current literature showed that there have been only 31 cases of omental teratomas described to date, and three of these cases had both dermoid cysts in the ovaries and teratomas in the omentum^(3)^. In this report, we present two patients with teratomas in the omentum, one a woman aged 34 years, and the other a postmenopausal woman aged 74 years; both patients’ dermoid cysts were surgically treated with ovarian cystectomy.

## CASE REPORT

**Figure 1:**
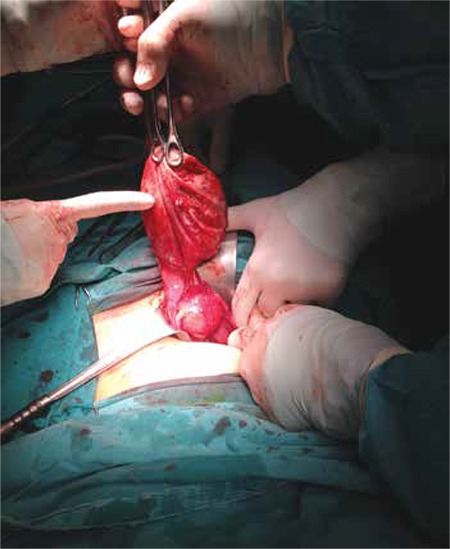
Parasitic dermoid cyst adhered to the uterus in surgical observation

Our first case was a nulliparous woman aged 34 years who had been married for six months, and was initially admitted to another centre because of symptoms of severe pelvic pain. A cystic mass was identified in the patient’s right adnexal area after an ultrasonographic (USG) assessment was conducted, and she was subsequently referred to the Gaziantep University Faculty of Medicine, Department of Gynecology and Obstetrics. The USG performed at our centre identified a 10 cm cystic mass in the right adnexal area with internal echogenic focus. The left ovary and uterus were normal, and the patient had a regular menstrual cycle. Preoperative assessment of cancer antigens (CA) 125 and 19-9 indicated values within normal range. Laparotomy was performed in January 2015 based on a preliminary diagnosis of ovarian dermoid cyst. The left ovary and oviduct were visible in a perioperative examination, but the right ovary and oviduct could not be observed. In the fundus of the uterus, we observed a 10 cm dermoid cyst surrounded by the omentum that had adhered to the uterus wall (Figure 1). After performing adhesiolysis, it was observed that the ovary on the fundus of the uterus lacked any feeding blood vessel, and the ovary tissue was excised together with the cyst. Pathology results confirmed a dermoid cyst.

**Figure 2:**
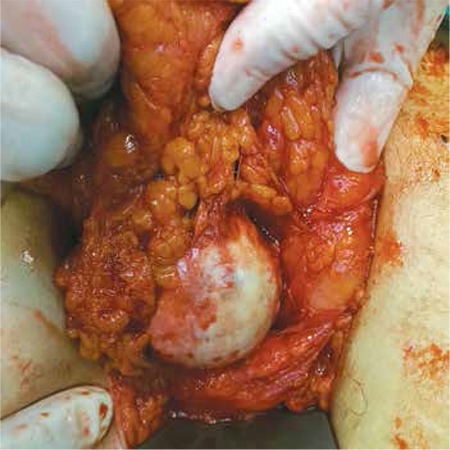
Parasitic dermoid cyst surrounded by the omentum in the surgical examination

Our second case was a postmenopausal woman aged 74 years who was initially admitted at another centre because of severe pelvic pain. After a dermoid cyst was suspected in the patient’s magnetic resonance ımaging (MRI) evaluation, she was referred to the Gaziantep University Faculty of Medicine, Department of Gynecology and Obstetrics. USG performed at our centre revealed atrophy in the uterus and bilateral ovaries. Preoperative assessment of CA 125 indicated normal values and CA 19-9 was 62 U/mL. A laparotomy was performed in February 2015 based on a preliminary diagnosis of ovarian dermoid cyst. A perioperative examination revealed an atrophic left ovary and oviduct, but the right ovary and oviduct could not be seen. The omentum was adhered to the abdomen, and contained a 5 cm dermoid cyst (Figure 2). The dermoid cyst was excised, and a partial omentectomy was performed along with a total hysterectomy and left salpingo-oophorectomy. The pathology results confirmed a dermoid cyst. In both cases, the macroscopic benign appearance of the mass, absence of ascites, and preoperative presumptive diagnosis of a parasitic dermoid cyst were considered and we did not send the specimen to frozen section.

## DISCUSSION

Teratomas generally develop from germs cells within the gonads. In the early stages of embryonic development, germs cells inside the yolk sack move towards the genital ridge by migrating along the hindgut. These germ cells, which are totipotent, develop into various types of tissues. The cells’ movement over the hindgut also indicates why teratomas can occur in a variety of locations^(4)^. The underlying mechanism for the development of teratomas in the omentum has not yet been clarified; however, there are three theories on the possible cause for these cysts’ occurrence and their diverse locations:

1. Omental teratomas might develop from germ cells that, for one reason or another, may have been displaced.

2. Omental teratomas might develop within a supernumerary ovary.

3. Omental teratomas might develop due to the self-amputation of a dermoid cyst in the ovary, and then become re-implanted into the omentum^(5)^.

Of the three mechanisms listed above, the third is likely to have been the underlying cause in our two cases. The dermoid cysts in our cases could have detached from their initial location after losing their blood supply, and then moved to another location where they re-vascularized and gained a new blood supply. There is also the possibility that previous surgical treatments might have left ovarian tissue remains that later became parasitic^(1)^. The omentum, which assumes an important role in the inflammatory defence mechanism of the intra-abdominal area, is the most common target for the cysts’ re-implantation^(6)^. In both of our cases, the dermoid cysts lacked any visible blood supply and vascularization, which suggests that they might have become detached from the ovary through self-amputation as a result of torsion. Although the clinical picture of parasitic dermoid cysts normally includes abdominal pain, it may also be associated with uncommon signs such as distension in the abdomen^(3)^. The only symptoms observed in our patients were severe pelvic pain. Teratomas of the omentum normally appear as palpable round and mobile masses in the abdomen. They can be located in the pelvis, where they might be mistaken for an adnexal mass, an upper abdominal mass, or a periumbilical mass. The location of omental teratomas might slightly change from one examination to another. One of our patients exhibited a mobile and palpable mass in the right pelvic area during her physical examination, whereas the other patient’s physical examination revealed no abnormal findings. A preoperative diagnosis of omental teratoma is often difficult. Diagnosis can be established using computerized tomography, MRI, and USG. However, a definite diagnosis usually requires histopathologic examination, which helps distinguish immature from mature teratomas. Although omental teratomas are benign tumours in the majority of cases, a number of malignant omental teratomas have also been reported(^7)^. Only three cases of immature teratoma have been reported in the English literature^(8)^. Immature teratomas, which are also known as nongerminomatous germ cell tumours, consist of primitive neuroectodermal, endodermal or mesodermal tissue. They have a higher susceptibility to metastasis compared with mature teratomas. In addition, age, sex, and location of the tumor can affect their aggressive behavior^(9)^. The management of these patients, including early stage disease, is chemotherapy after surgery has been performed. The bleomycin-etoposide-platinum combination is the most recommended protocol for all stages of nonseminomatous germ cell tumours^(10)^. Standard treatment involves laparoscopy and laparotomy, with tumour excision accompanied by partial omentectomy; this is sufficient in most cases^(5)^. The treatment approach against omental teratomas is determined based on the tumour’s level of maturity, with full surgical excision providing adequate treatment against mature teratomas.

In conclusion, the greater part of the omental teratomas in our patients appear to have developed from the self-amputation of adnexal cysts in the ovary, followed by reimplantation into the omentum. Although ultrasonography and computerized tomography might aid diagnosis, determining the exact location of intraabdominal ovarian parasitic cystic teratomas with these methods can prove to be challenging. For this reason, color flow Doppler is often used to assist localization. In the event that laparotomic exploration reveals a unilateral lack of adnexa in a patient, the abdominal cavity should be explored for tumors that might have self-amputated and re-implanted in this area. In patients with such unusual unilateral absence of adnexa, gynecologic surgeons should consider the possibility of parasitic dermoid cysts that have re-localized in extragonadal areas during differential diagnosis. Awareness among gynecologic surgeons of such masses may help prevent misdiagnosis, delayed surgery, or the use of wrong surgical approaches.
